# Deep Learning Models for Automated Assessment of Breast Density Using Multiple Mammographic Image Types

**DOI:** 10.3390/cancers14205003

**Published:** 2022-10-13

**Authors:** Bastien Rigaud, Olena O. Weaver, Jennifer B. Dennison, Muhammad Awais, Brian M. Anderson, Ting-Yu D. Chiang, Wei T. Yang, Jessica W. T. Leung, Samir M. Hanash, Kristy K. Brock

**Affiliations:** 1 Department of Imaging Physics, The University of Texas MD Anderson Cancer Center, Houston, TX 77030, USA; 2 Department of Breast Imaging, The University of Texas MD Anderson Cancer Center, Houston, TX 77030, USA; 3 Division of Diagnostic Imaging, The University of Texas MD Anderson Cancer Center, Houston, TX 77030, USA; 4 Department of Clinical Cancer Prevention, The University of Texas MD Anderson Cancer Center, Houston, TX 77030, USA; 5 Department of Radiation Medicine and Applied Sciences, University of California San Diego, San Diego, CA 92093, USA; 6 Department of Radiation Physics, The University of Texas MD Anderson Cancer Center, Houston, TX 77030, USA

**Keywords:** full-field digital mammograms (FFDM), digital breast tomosynthesis (DBT), deep learning (DL), synthesized 2D images

## Abstract

**Simple Summary:**

The DL model predictions in automated breast density assessment were independent of the imaging technologies, moderately or substantially agreed with the clinical reader density values, and had improved performance as compared to inclusion of commercial software values.

**Abstract:**

Recently, convolutional neural network (CNN) models have been proposed to automate the assessment of breast density, breast cancer detection or risk stratification using single image modality. However, analysis of breast density using multiple mammographic types using clinical data has not been reported in the literature. In this study, we investigate pre-trained EfficientNetB0 deep learning (DL) models for automated assessment of breast density using multiple mammographic types with and without clinical information to improve reliability and versatility of reporting. 120,000 for-processing and for-presentation full-field digital mammograms (FFDM), digital breast tomosynthesis (DBT), and synthesized 2D images from 5032 women were retrospectively analyzed. Each participant underwent up to 3 screening examinations and completed a questionnaire at each screening encounter. Pre-trained EfficientNetB0 DL models with or without clinical history were optimized. The DL models were evaluated using BI-RADS (fatty, scattered fibroglandular densities, heterogeneously dense, or extremely dense) versus binary (non-dense or dense) density classification. Pre-trained EfficientNetB0 model performances were compared using inter-observer and commercial software (Volpara) variabilities. Results show that the average Fleiss’ Kappa score between-observers ranged from 0.31–0.50 and 0.55–0.69 for the BI-RADS and binary classifications, respectively, showing higher uncertainty among experts. Volpara-observer agreement was 0.33 and 0.54 for BI-RADS and binary classifications, respectively, showing fair to moderate agreement. However, our proposed pre-trained EfficientNetB0 DL models-observer agreement was 0.61–0.66 and 0.70–0.75 for BI-RADS and binary classifications, respectively, showing moderate to substantial agreement. Overall results show that the best breast density estimation was achieved using for-presentation FFDM and DBT images without added clinical information. Pre-trained EfficientNetB0 model can automatically assess breast density from any images modality type, with the best results obtained from for-presentation FFDM and DBT, which are the most common image archived in clinical practice.

## 1. Introduction

Female breast cancer is the most common cancer diagnosed worldwide representing 11.7% of all cancers from both sexes while representing the fifth deadliest cancer for women with a mortality rate of 6.9% in 2020 [1]. Thanks to technological and populational health advancements, breast cancer can be detected and diagnosed earlier to offer optimal treatment options, reducing the mortality rate by 38–60% [2,3,4]. However, mammography cancer detection can be hampered by dense breast parenchyma. While high breast density is also a risk factor for breast cancer, sensitivity of full-field mammography screening is lower for subjects with high breast density, reducing the chance of early optimal treatment [5].

Personalized screening strategies incorporating risk assessment, utilization of appropriate radiologic modalities, and better image analysis are needed to improve breast cancer detection in women with dense breasts [6,7]. To achieve this goal, breast density assignment needs to be standardized, reproducible, and preferably automated [8]. Historically, breast density is visually assessed on mammography during image interpretation in the usual clinical workflow, but is prone to inter- and intra-observer variabilities, especially for adjacent density categories (e.g., scattered fibroglandular densities and heterogeneously dense) [9,10]. To promote standardization and consistency between radiologists, computer software tools have been developed and proposed to assess breast density directly from for processing or for-presentation mammograms. While commercially available tools may aid in breast density categorization, visual assessment continues to be reported to provide the best performance [11].

More recently, convolutional neural network (CNN) models have been proposed to automate the assessment of breast density in an efficient and robust manner for large scale screening [12,13,14]. CNN models also have been proposed for breast cancer detection or risk stratification using clinical history with full field mammograms or multi-parametric MRI, [15,16,17,18] showing promising results. Additionally, CNN-based applications have been studied for purpose of risk assessment [19,20]. Despite these early results suggesting clinical roles for CNN-based applications, deep learning-based approaches need to be further investigated and validated prior to implementation in clinical practice.

This study investigates the role of state-of-the-art CNN architectures for breast density estimation using multiple mammographic types. We explored the concordance between radiologists, commercial breast density estimation software, and DL models trained on different mammographic types. The models were evaluated with and without clinical participant information. Breast density was estimated using two approaches: Breast Imaging Reporting and Data System (BI-RADS) four breast density versus binary (non-dense or dense) categorization.

## 2. Materials and Methods

### 2.1. Data

The Institutional Review Board approved this study and waived informed consent. We performed a retrospective analysis of imaging and health questionnaire data from 5032 participants enrolled in a prospective breast cancer screening cohort at a single academic institution. The demographic composition of the cohort is representative of the female population of a large metropolitan area served by the institution. The imaging dataset contained a total of 120,000 for processing FFDM, for presentation FFDM, synthesized 2D and for-presentation digitally reconstructed DBT craniocaudal (CC) and mediolateral oblique (MLO) views of both breasts acquired between 2017 and 2021. Each participant had up to three consecutive routine bilateral mammographic exams included in the analysis. At each screening the participants completed a detailed questionnaire. A combination of all questions answered by each participant at each screening timepoint resulted in a single consolidated questionnaire that was used for the study. The consolidated questionnaire comprised twenty-one questions regarded as potentially pertinent to breast density and were included in the analysis (Table S1, supplementary material). Each mammographic study was assigned one of four BI-RADS breast density categories of fatty (A), scattered fibroglandular densities (B), heterogeneously dense (C), or extremely dense (D) by a breast radiologist using the FFDM images. Table S1 contains the demographics of the study population, the 21 questions included in the analysis, the number of images per modality, and the BI-RADS breast density of the cohort per year of screening. All mammograms were acquired using Selenia Dimensions Mammography System (Hologic, Marlborough, MA, USA). All synthesized 2D mammograms were generated using the Hologic C-ViewTM algorithm. All images were acquired as standard acquisition imaging parameters as shown in supplementary material Table S2.

1000 of the 5032 participants have been previously reported using 4394 matched pairs of for processing and for presentation FFDM [21]. This prior study developed a DL-based approach to recreate for-processing FFDM from for-presentation mammograms. The 5032 cases were consecutive cases enrolled into the MERIT screening study with no history of breast cancer, no history of treatment for any invasive cancer within the last five years, 25–80 years of age, and no breastfeeding within the last six months.

### 2.2. Convolutional Neural Networks (CNN)

#### 2.2.1. Model Architectures

This study investigated the EfficientNetB0 architecture with pre-trained ImageNet features [22]. The EfficientNetB0 architecture is composed of 7 layers including convolution and inverted residual blocks with squeeze and excitation optimization and a 20% rate dropout layer for regularization. EfficientNet architectures were initially developed to provide the best compromise between Top1 and Top5 accuracy on ImageNet, and network width, depth, resolution, number of parameters, and training time. This architecture was selected for this study based on the excellent compromise between performance and model size, allowing the combination of multiple sub models per mammographic image type. Output of the EfficientNet model was a 1 × 1 convolution, normalization layer and swish activation layers with output size of 16 × 16 × 1280 (x × y × Nfilters). The 2D and 3D networks were trained with batch and group (n = 32) normalization layers, respectively. For the classification part, a 2D global average pooling was used for all 2D input models (FFDM for-processing, FFDM for-presentation, synthesized 2D) to flatten the convolution output as a vector of 1280. For the 3D model using DBT, the batch size and slice dimensions were squeezed together to extract 2D features on each of the tomosynthesis slices. The 2D features in the 3D space were flatten together using a 3D global average pooling. The top part of the architecture was composed of *N* dense layers with U units with swish activation, no dropout, and a final softmax dense layers with 4 units for categorical classification. The values of N/U were 0/0, 1/512, 2/256, and 0/0, for the for processing, for presentation, synthesized 2D and DBT mammograms models, respectively. For the multimodal model, each modality sub-model was frozen using the pre-trained features from the independent models, only the classification part of the model was optimized.

To estimate the breast density using the participant’s health history and measurements as input, a total of 21 features were extracted from the questionnaires and converted as floating or categorical variables. Subject features were used to train a CNN of five layers combining dense layers of 32 units, dropout layers of 5%, and swish activations followed by a softmax dense layers with 4 units for categorical classification.

**Figure 1:**
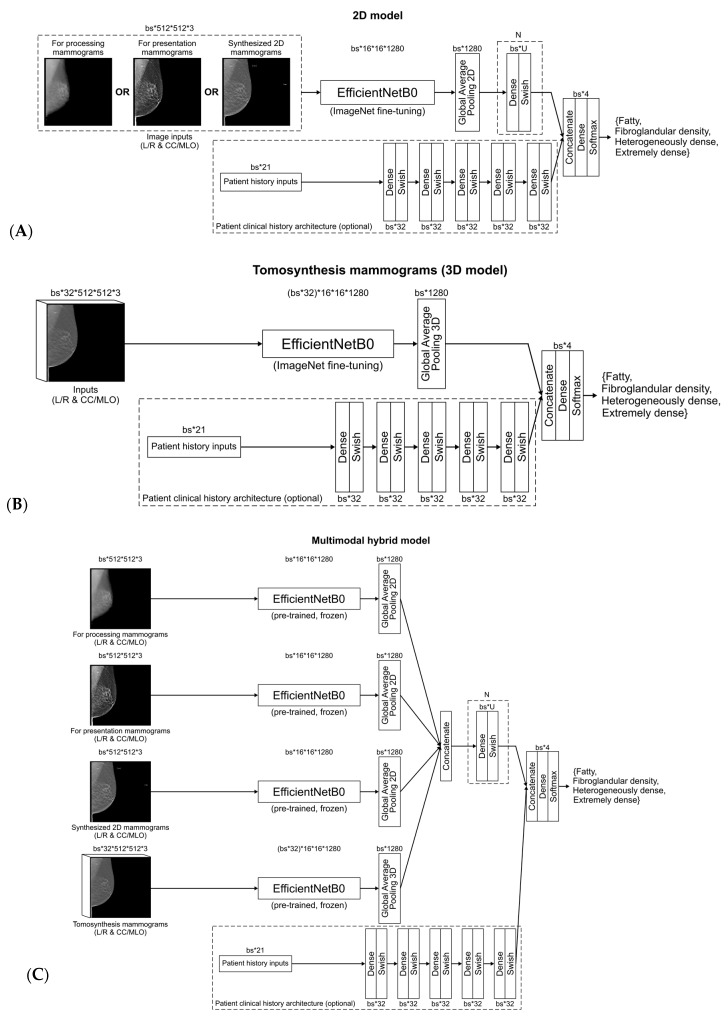
Convolutional neural network model for (**A**) 2D mammograms (i.e., for processing, for presentation, synthesized 2D), (**B**) digital breast tomosynthesis, (**C**) multimodal mammograms, with clinical history.

Figure 1A–C summarizes the different model architectures. A total of 11 CNN models were defined in this study: 1 feature-based only model using the questionnaire, 4 models (1 per mammographic type) without inclusion of clinical history (for processing FFDM, for presentation FFDM, synthesized 2D, and DBT), 4 models (1 per mammographic type) with inclusion of clinical history (for processing FFDM, for presentation FFDM, synthesized 2D, and DBT), and 2 models including all modalities (1 without clinical history and 1 with clinical history).

#### 2.2.2. Training Strategy

The data were randomly separated into groups of mammograms from 3044, 993, and 995 subjects for the training, validation, and withheld test datasets, respectively. Random distribution of subjects was stratified based on demographics (Table S1, supplementary material). Multiple screenings and images from an individual participant were not distributed among training, validation, and test datasets. Each of the 11 models listed above was trained with both views (i.e., CC and MLO) and both sides (i.e., left and right). The background of the images was removed to avoid biased feature extraction outside the breast [21]. All image intensities were Z standardized and normalized between 0–255. The images for the 2D and 3D networks were resized to 512 × 512 and 512 × 512 × 32 dimensions, respectively, with in-plane ratio preservation. The 3D images were resized in 2 steps, first by resizing the Z direction to 32 slices with a linear interpolator while keeping the XY original size, second by resizing only the XY planes to 512 × 512 while keeping the size ratio using image padding.

Each model was trained to output a categorical probability distribution at inference on a single mammogram (e.g., left, right, CC or MLO views) representing the probability of different BI-RADS density categories. To avoid gradient washing and fine tune the ImageNet pre-trained model weights and features, respectively, we progressively trained the model into 7 stages by unfreezing the weights of the convolution block layers using Adam optimizer [23] with the categorical cross entropy loss function using a stable learning rate of 0.0001 that was decreased by a factor of 4 at each stage of the training strategy. To improve model generalization, all models were trained with data augmentation of random left-right and up-down flips. The 2D network models were also trained with rotation ranging between 0–15 degrees, translation ranging between 0–40 mm, scaling ranging from 0–20%.

No class imbalance adaptation was considered using weighted loss or oversampling in our training strategy. However, training subject and class distributions shuffling was performed at data creation, cache and before each epoch to improve stability, training time and validation accuracy. For 2D and 3D network inputs, the batch size values were 32 and 1, respectively. For the multimodal model, batch size value was 1.

For the clinical history dataset, missing answers from participant were replaced by zeros. To avoid overfitting, dropout layers with rate of 5% were included with clinical history CNN to simulate random missing inputs from participant’s answer. For the clinical history CNN, the batch size value was 64. No data augmentation was performed on the history input.

### 2.3. Evaluation

To evaluate inter-observer uncertainty in the test dataset, a total of 7 fellowship trained breast radiologists with 5–22 years of experience defined the BI-RADS breast density categories on the baseline screening (year 0) for-presentation FFDM of 1000 participants evaluated per breast, for a total of 2000 breasts. For this task, the FFDMs were randomly divided into 10 groups of 200 subjects. Each group was evaluated by 3 radiologists, which resulted in 6000 annotated studies. Inter-observer variability was compared per reading group and by pair of observers for every group using the Fleiss’ Kappa score with and without multiple raters, respectively. Percentage of agreement was also reported. The Kappa score (κ) ranges from −1 and 1 where κ < 0 represents no agreement, 0.01 < κ < 0.20 slight agreement, 0.21 < κ < 0.40 fair agreement, 0.41 < κ < 0.60 moderate agreement, 0.61 < κ < 0.80 substantial agreement, and 0.1 < κ < 1.00 almost perfect agreement. Out of the 1000 subjects, analysis was gathered on the 995 subjects from the test dataset.

#### 2.3.1. Commercial Tool Evaluation

A commercially available software tool, Volpara Density Algorithm 3.4.1 (Volpara Health Technologies, Wellington, New Zealand), was used on 960 out of the 995 test subjects to assess breast density on FFDMs. Volpara reports the patient’s breast density as being the highest breast density from both sides. The performance of Volpara in assigning BI-RADS was compared with the ground truth densities, inter-observer variability and DL model predictions.

#### 2.3.2. Model Evaluation

Model evaluation was reported on the validation and withheld test datasets using true positive (TP), false positive (FP), categorical accuracy, precision, recall, F1-score per class, receiver operating characteristic (ROC), area under the curve (AUC), percentage of agreement, and Kappa metrics between the clinical reader density class and model predicted probability of BI-RADS, and binary (non-dense [categories A and B] versus dense [categories C and D]) breast densities. The results were averaged between the available views of each breast screening. To summarize the model performance per class, confusion matrices were generated for categorical BI-RADS and binary breast densities. Binary breast density was estimated to evaluate model uncertainty between neighbor categories (e.g., scattered fibroglandular densities and heterogeneously dense) versus non-dense and dense categories. To assess the model interpretability in classification decision, both Gradient-weighted Class Activation Mapping (Grad-CAM) [24] and Integrated Gradients [25] activation maps were generated. A continuous BI-RADS score was estimated by weighting the category values by their respective probabilities. Precision, recall, and AUC were computed using scikit-learn python library with the “weighted” average parameter to account for multiple categories classification and label imbalance. The other metrics were computed using TensorFlow 2.4 and Tensorflow Addons python libraries. The TP and FP metrics were computed as being the sum of diagonal and non-diagonal values from the confusion matrix, respectively. F1 score values were reported per class.

## 3. Results

### 3.1. Expert Evaluation

The median (min-max) Fleiss’ Kappa scores assessing the 3 radiologists’ agreement per group (i.e., a total of 7 radiologist assessing randomly distributed 10 groups of 200 patients) and per density were 0.47 (0.21–0.87), 0.28 (−0.05–0.60), 0.55 (0.41–0.67), and 0.34 (0.13–0.70) for fatty, scattered fibroglandular densities, heterogeneously dense, or extremely dense breasts BI-RADS categories, respectively. Extracting agreement between pair of observers on the same subject, Fleiss’ Kappa scores and percentage of agreement ranged between −0.05–0.76 and 31–87%, respectively. Two observers with the lowest agreement within the group had an average Fleiss’ Kappa score and percentage of agreement of 0.32–0.36 and 57–59% compared to 0.40–0.57 and 60–65% for the 5 others, respectively. Reducing the number of categories to 2 (non-dense or dense), the Fleiss’ Kappa score and percentage of agreement increased in average by 0.20 and 21%.

**Figure 2:**
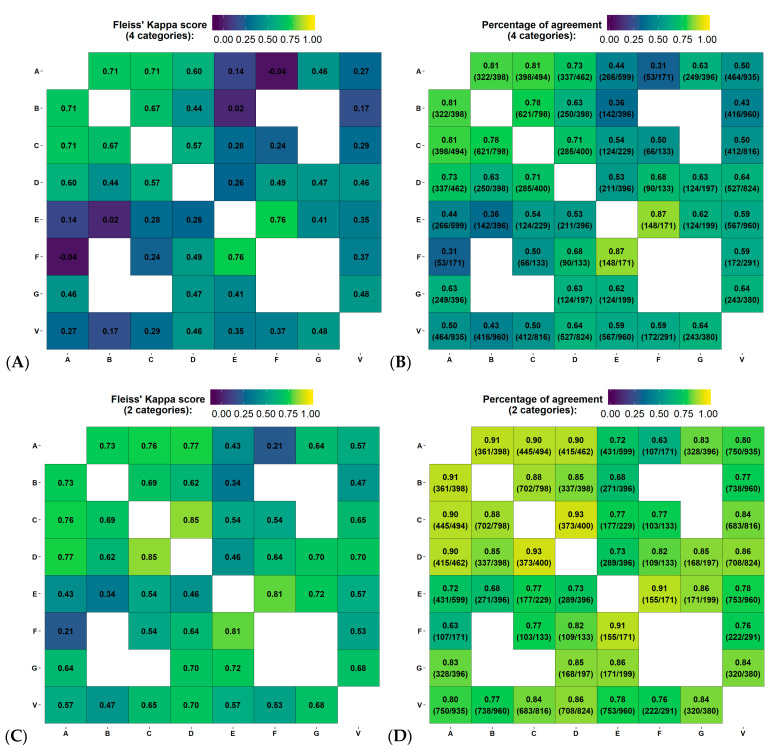
Variability in the estimation of breast density using Fleiss’ Kappa score and percentage of agreement between pair of observers, for all reading groups. (**A**–**D**) represent the agreement between 4 (i.e., fatty, scattered fibroglandular densities, heterogeneously dense or extremely dense) and 2 categories (non-dense or dense), on the first screening year of the test dataset. Physician experience in years of service: A = 8, B = 13, C = 8, D = 9, E = 5, F = 20 and G = 22 years. V = Volpara version 17.

**Figure 3:**
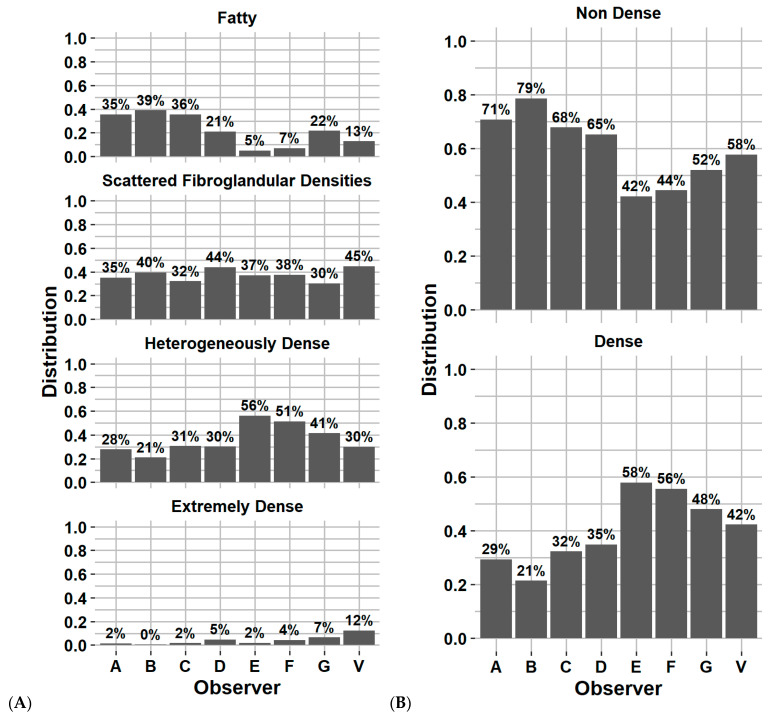
Distribution of breast density category assignment by each observer and Volpara software for (**A**) 4 categories and (**B**) 2 categories, on the first screening year of the test dataset. Physician experience in years of service: A = 8, B = 13, C = 8, D = 9, E = 5, F = 20 and G = 22 years. V = Volpara Density Algorithm 3.4.1 (Volpara Health Technologies, Wellington, New Zealand).

Figure 2A–D represents the Fleiss’ Kappa score and percentage of agreement as confusion matrices for each available pair of observers. Figure 3A,B reports the distribution of breast density assignment per observer for 4 and 2 categories.

### 3.2. Commercial Software Evaluation

Figure 2A–D reports the Fleiss’ Kappa scores and percentage of agreement as confusion matrices for Volpara compared to each observer from the inter-observer variability study on the test dataset. The Fleiss’ Kappa score and percentage of agreement ranged from 0.17–0.48 and 43–64%, respectively. The average Fleiss’ Kappa score and percentage of agreement were 0.34 and 56%, respectively, corresponding to fair agreement. Using binary classification, the metrics increase by 0.25 and 25%, respectively, corresponding to moderate agreement. Figure 3A,B reports the distribution of breast density assignment by Volpara for 4 and 2 categories compared to each observer.

Comparing the Volpara classification with the clinical reader density class (i.e., used to train the DL models), the Fleiss’ Kappa and percentage of agreement were 0.33 and 58% for 4 categories and 0.54 and 77% for 2 categories, respectively, on 960/995 test subjects using year 0 screening.

### 3.3. DL Model Evaluation

Table 1 and Table 2 report the accuracy of each DL model per mammographic type with and without including subject history on the test dataset for BI-RADS and binary classifications, respectively. Similarly, Tables S3 and S4 (supplementary material) report the accuracy for the validation dataset.

**Figure 4:**
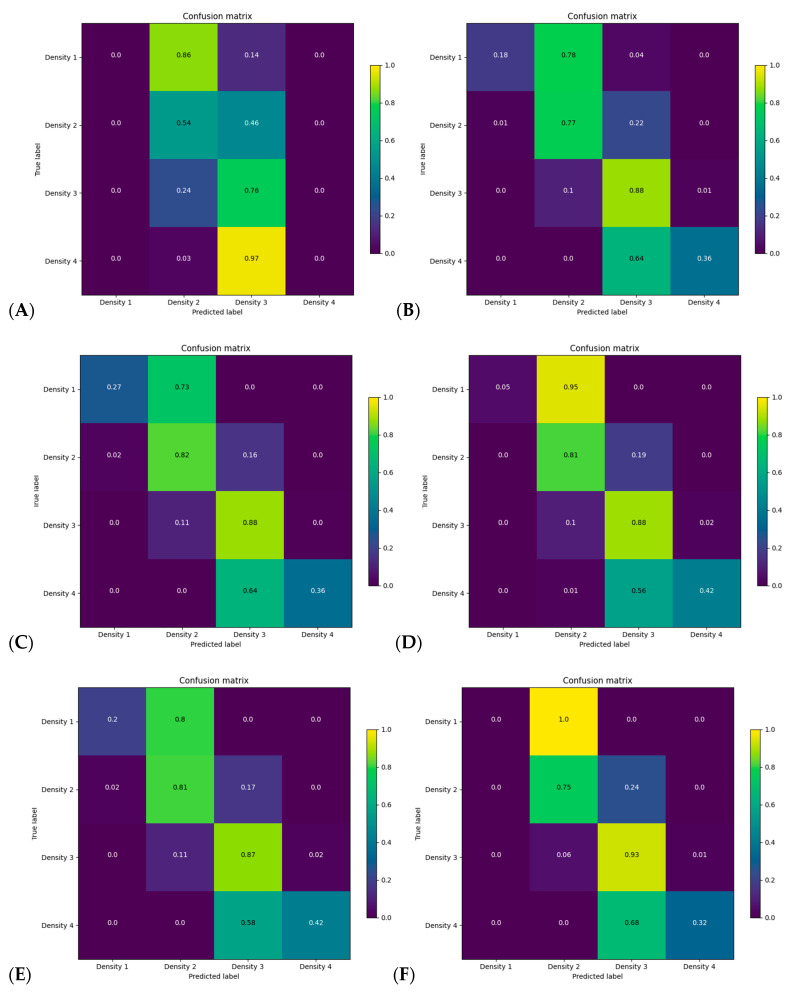
Confusion matrix for categorical breast density for each model on every screening year of the test dataset without including clinical history for (**A**) history, (**B**) for processing, (**C**) for presentation, (**D**) synthesized 2D, (**E**) digital breast tomosynthesis, mammograms and (**F**) all modalities models.

**Figure 5:**
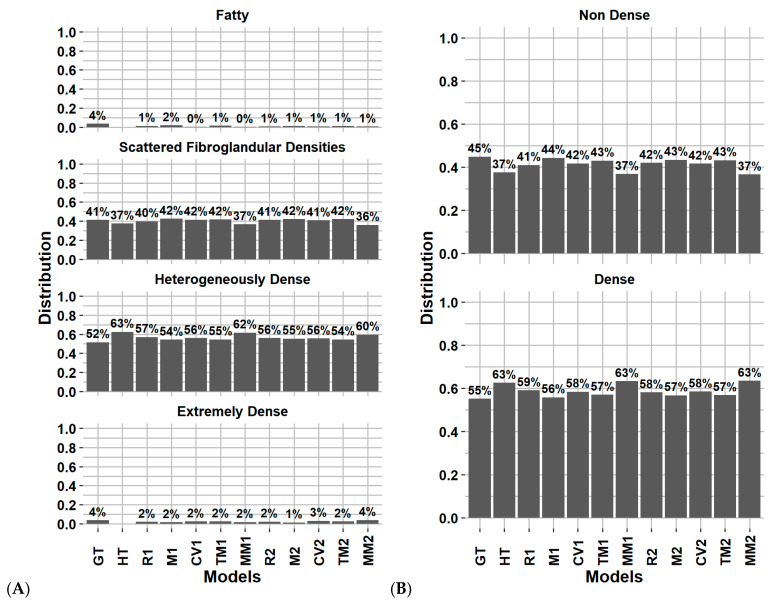
Distribution of breast density category assignment for the ground truth and DL models on for (**A**) 4 categories and (**B**) 2 categories, on the test dataset considering every screening (except H). Abbreviations: GT: ground truth (clinical reader), HT: history model, R1: for processing, M1: for presentation, CV1: synthesized 2D, TM1: digital breast tomosynthesis, MM1: Multi model, 2: include clinical history.

Figure 4 reports the confusion matrix of BI-RADS classification for each model without including subject history. Figure 5A,B reports the distribution of breast density assignment per model for 4 and 2 categories. Figures S1 and S2 (supplementary material) report the confusion matrices and distribution on the validation dataset.

Using the for-presentation FFDM without history model, Figure 4C reports that the model uncertainty was mostly present in the fatty and extremely dense categories, with 27% and 36% of these classes being correctly classified, respectively. The model misclassified 73% of the fatty breast to scattered fibroglandular densities and 64% of the extremely dense breast to heterogeneously dense. Within BI-RADS B and C classes, a total of 16% and 11% of the subjects were misclassified as being dense compared to non-dense and non-dense compared to dense, respectively.

For comparison between subgroup inter-observer, Volpara, and each DL model, Figure S3 (supplementary material) reports the breast density distribution for the 4 and 2 categories classification using the year 0 screening on the 995 subjects from the test dataset. On the year 0 screening of the test dataset, the Fleiss’ Kappa and percentage of agreement range of the image-based DL models were 0.61–0.66 and 79–82% for 4 categories and 0.70–0.75 and 86–88% for 2 categories, respectively.

**Figure 6:**
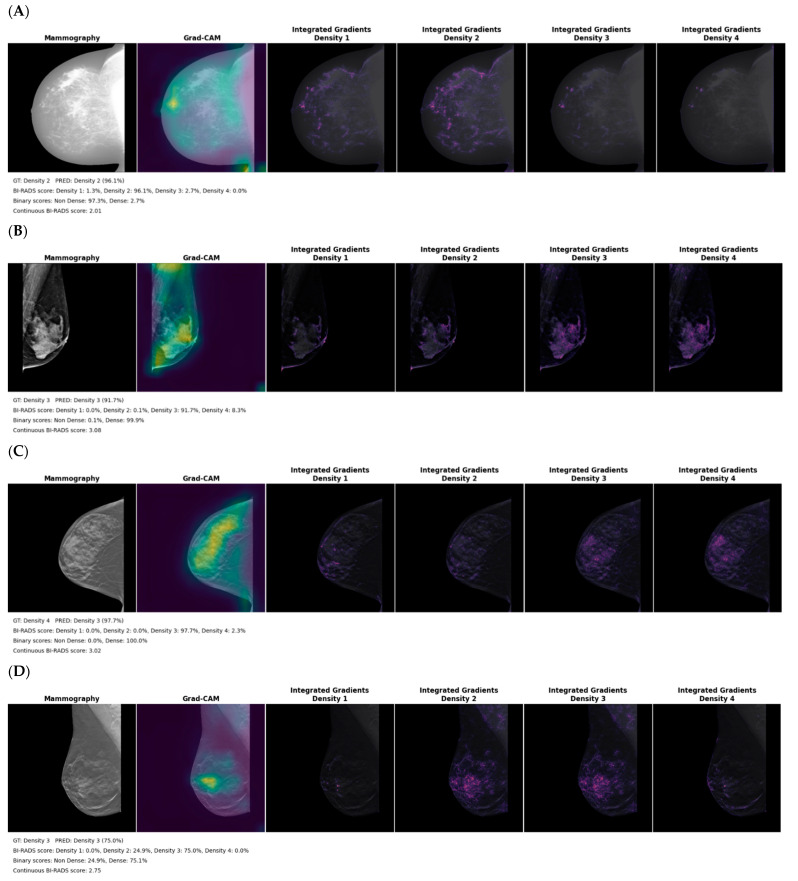
Grad-CAM and integrated gradients activation maps for the (**A**) for processing (**B**) for presentation (**C**) synthesized 2D and (**D**) digital breast tomosynthesis mammography models on the test dataset. The legend includes the ground truth (GT) clinical reader density class, predicted (PRED) density, the probability for the 4 classes, the summed probability for binary output and the continuous BI-RADS score.

Figure 6 represents the Grad-CAM and integrated gradients activation maps for each single input image model. Each example represents the normalized breast image with an overlay of the Grad-CAM and integrated gradients activation maps. Each integrated gradients activation map represents the extracted features in the image that explain each class.

## 4. Discussion

In this study, we investigated the performance of fine-tuned state of the art DL architecture for breast density assessment using 4 mammography image types individually and in combination, with and without clinical history. The models were validated on a completely withheld test dataset including 6300 to 12,000 breast images from 995 subjects. The performance of the DL models was also compared with inter-observer variability, quantified from the density estimated by 7 clinical radiology experts, and a commercially available tool in the test dataset. To our knowledge, this study is the first to perform an exhaustive evaluation of DL-based breast density assessment on all mammography types.

Our results demonstrate that the models with the highest categorial accuracy and Kappa score for BI-RADS density classification were the for-presentation FFDM and DBT models without inclusion of clinical history. For the binary classification, the for-presentation FFDM model was the most accurate in classifying subjects’ density as being non-dense or dense. The model using only the clinical history to predict the breast density had only a fair agreement with the clinical reader density class (range 0.26–0.33). Including clinical history with the image features in the classification step of the DL models slightly decreased the accuracy. This could be in part due to the fact that a combined questionnaire was used for each participant, and the answers might have changed over the course of the observation, reducing its accuracy for each separate timepoint (years 1, 2 and 3). In addition, the 21 questions selected for potential impact on breast density. Overall, only a minimal difference was observed in the accuracy reported by every DL model regarding modality and number of training images. All models provided a substantial agreement with the clinical reader density class (range 0.61–0.75).

Lower agreement between model prediction and expert ground truth was observed with the for- processing mammography; this could be explained by the larger differences in intensity between subjects as well as the heavily skewed intensity histogram within the breast that made standardization challenging. While the for-processing mammography model extracts features within dense breast tissue (Figure 6A), the activation map shows a systematic strong focus on the lower corner of the image that suggests sensitivity of the model to image padding, frailness to differentiate BI-RADS categories, or even limited transferability of the ImageNet pretrained features on that modality.

The findings from this study suggest that combining modalities or including subject history do not improve accuracy compared to the per-modality model. They also suggest that no modality is superior to another in terms of assessing breast density. However, we did observe differences in misclassification of adjacent classes (Figure 4 and Figure S1, supplementary material), with the best models to distinguish 86% of non-dense and 94% of the dense categories being the for- presentation FFDM and DBT without history models, respectively. This is potentially clinically useful, since these image types are the ones most archived in clinical practice and are easily accessible for density evaluation. The results show that no modality is superior to another in terms of assessing breast density. Therefore, in the future real-time implementation of our research in the clinical environment will act as an aided tool for the radiologist to perform breast density evaluation more effectively and efficiently using AI models irrespective of image modalities. Compared to the recent published study by Lehman et al. [13] that investigated a DL model for automatic breast density evaluation for 4 BI-RADS or binary categories, we obtained similar Kappa scores (0.66 vs. 0.67, ours vs. theirs, respectively) but with a higher BI-RADS classification for the scattered fibroglandular and heterogeneously dense categories. For a binary breast density classification and comparing to their model development validation (test dataset), the percentage of well classified non-dense and dense categories were 86% vs. 89% and 90% vs. 84% (ours vs. theirs, respectively, using for-presentation FFDM), respectively.

Our study also reports high variability in assessing breast density of 1000 subjects by groups of three radiologists. The reported agreement between observers showed larger uncertainty in assigning the scattered fibroglandular densities and extremely dense categories, with averaged Fleiss’ Kappa scores of 0.28 and 0.34. These results are similar to previously reported data, with a Kappa score of 0.48 (moderate agreement) described by Gweon et al. [26] To evaluate a clinically available automated breast density tool, we also computed the breast density on 960/995 test subjects using a commercially available algorithm, Volpara Density Algorithm 3.4.1, that showed an averaged Kappa score of 0.34 and 0.33 (fair agreement) with the inter-observer study and clinical reader ground truth at baseline screening, respectively. Reducing the categories to non-dense and dense, Kappa score and percentage of agreement at baseline screening with clinical reader ground truth increased to 0.54 and 77%, respectively. This was close to the value reported in a previous study by Brandt et al. evaluating Volpara and reporting an average Kappa score of 0.46 [27].

During the CNN ablation study, we investigated two strategies to compensate class imbalance by integrating the training dataset class imbalance in the categorical cross entropy loss function or by oversampling minority classes in our dataset pipeline. However, we did not observe any improvements but reduced validation accuracy, showing that the model was not able to learn and converge properly. To compensate for class imbalance in the best way possible, we heavily shuffled the training dataset before each epoch and chose a large batch size for the 2D models to show a large number of examples per class to the model.

Our study has several limitations. First, the dataset comes from one clinical center, although our patient population reflects the demographic diversity of the female population served by the center (Table S1, supplementary material). Second, all images were acquired using one type of mammography acquisition system and the third, ground truth was determined by a single clinical radiologist as part of clinical process. The clinical radiologists had access to the Volpara results for all cases when making their clinical decision. We randomly split the data into training, validation and withheld test datasets with subject demographics stratification. However, we observed a slight decrease of 0.05 Kappa score in the accuracy on the test dataset compared to the validation dataset (Table 1 and Table S3). Third, the different number of images per modality group and dataset hampers direct comparison of the proposed per-modality models. However, this reflects the challenge of collecting a large amount of data per subject in a clinical workflow to train large DL models with different modalities. In the future, the results of this study will need to be confirmed with external data from other clinical centers with different imaging systems. The results show that no modality is superior to another in terms of assessing breast density. Therefore, in the future real-time implementation of our research in the clinical environment will act as an aided tool for the radiologist to perform breast density evaluation more effectively and efficiently using AI models irrespective of image modalities.

The lack of improvement of model performance with clinical information in our study may be due to the fact that a consolidated questionnaire combining multiple timepoints was used, limiting the per time point accuracy of the information. In addition, we used questions with variable direct relevance to breast density. Using a more focused historical information to include the data traditionally associated with breast density, such as age, menstrual status, body mass index and exogenous hormone intake, may prove useful for improving DL model performance [28,29]. The next step of this study will be to further evaluate the DL model on an external test dataset, potentially using different image acquisition systems, as well as deploying the proposed models in a clinical workflow to gather clinical expert feedback from automated assessment of breast density and back-propagated activation map overlaid on the breast images.

## 5. Conclusions

DL models can be used to automatically assess breast density using images from any mammographic modality or image type. For-processing images in our database did not improve the model performance in our study, which suggests that routine storage of this data may not be needed for ML/DL studies. The best model performance was achieved on for-presentation FFDM and DBT images, which are the most common mammography image types archived in clinical centers. A set of 21 subject history questions chosen for this study deteriorated model performance, which may potentially be improved with a more focused choice of questions to include in the model. Further evaluation and cross-institutional model validation are needed before a multi-modality automated breast density model could be reliably deployed for multiple users.

## Figures and Tables

**Table 1:** Model accuracy on their respective test dataset for all screening years for 4 BI-RADS categories.

Model	Include History	Metric										
		TP	FP	Cat. Acc.	Precision	Recall	AUC	F1 per Class				Kappa
								1	2	3	4	
History	N/A	614	379	0.62	0.57	0.62	0.70	0.00	0.55	0.70	0.00	0.26
For processing mammography	No	1458	390	0.79	0.78	0.79	0.91	0.29	0.78	0.84	0.47	0.61
	Yes	1458	390	0.79	0.78	0.79	0.91	0.18	0.78	0.84	0.49	0.61
For presentation mammography	No	2067	461	0.82	0.81	0.82	0.93	0.36	0.81	0.86	0.50	0.66
	Yes	2063	465	0.82	0.81	0.82	0.93	0.31	0.81	0.86	0.47	0.66
Synthesized 2D mammography	No	1512	353	0.81	0.81	0.81	0.93	0.10	0.81	0.85	0.51	0.65
	Yes	1505	360	0.81	0.80	0.81	0.92	0.19	0.80	0.85	0.51	0.64
Digital breast tomosynthesis	No	1282	304	0.81	0.80	0.81	0.92	0.27	0.80	0.85	0.51	0.65
	Yes	1287	299	0.81	0.80	0.81	0.92	0.29	0.81	0.85	0.52	0.65
All modality	No	1058	260	0.80	0.77	0.80	0.92	0.00	0.78	0.86	0.45	0.63
	Yes	1059	259	0.80	0.79	0.80	0.92	0.11	0.78	0.86	0.65	0.64

**Table 2:** Model accuracy on their respective test dataset for all screening years for 2 categories (Non-dense and dense).

Model	Include History	Metric								
		TP	FP	Cat. Acc.	Precision	Recall	AUC	F1 per Class		Kappa
								Non-Dense	Dense	
History	N/A	669	324	0.67	0.67	0.67	0.73	0.60	0.72	0.33
For processing mammography	No	1580	268	0.85	0.86	0.85	0.94	0.83	0.87	0.70
	Yes	1581	267	0.86	0.86	0.86	0.94	0.83	0.87	0.71
For presentation mammography	No	2218	310	0.88	0.88	0.88	0.95	0.86	0.89	0.75
	Yes	2211	317	0.87	0.87	0.87	0.95	0.86	0.89	0.75
Synthesized 2D mammography	No	1625	240	0.87	0.87	0.87	0.95	0.85	0.89	0.74
	Yes	1625	240	0.87	0.87	0.87	0.95	0.85	0.89	0.74
Digital breast tomosynthesis	No	1384	202	0.87	0.87	0.87	0.95	0.85	0.89	0.74
	Yes	1384	202	0.87	0.87	0.87	0.94	0.85	0.89	0.74
All modality	No	1148	170	0.87	0.88	0.87	0.95	0.84	0.89	0.73
	Yes	1142	176	0.87	0.87	0.87	0.95	0.83	0.89	0.72

## Data Availability

The data presented in this study are available in this article and supplementary material.

## Supplementary Materials

The following supporting information can be downloaded at: https://www.mdpi.com/article/10.3390/cancers14205003/s1, Figure S1: Confusion matrix for binary breast density for each model on every screening year of the test dataset without including patient history for (A) history, (B) for processing, (C) for presentation, (D) Synthesized 2D, (E) digital breast tomosynthesis, mammograms and (F) all modalities models; Figure S2: Distribution of breast density category assignment for the ground truth and DL models on for (A) 4 categories and (B) 2 categories, on the validation dataset considering every screening (except H); Figure S3: Distribution of breast density category assignment for the ground truth and DL models on for (A) 4 categories and (B) 2 categories, on the test dataset considering the year 0 screening; Table S1: Participant demographics in training, validation and test datasets of a total 5032 participants randomly separated in 3044, 993, and 995 subjects; Table S2: Image parameters in training, validation and test datasets; Table S3: Model accuracy on their respective validation dataset for all screening years for 4 BI-RADS categories; Table S4: Model accuracy on their respective validation dataset for all screening years for 2 categories (Non-dense and dense).
