# Supplementary material for: Deep Learning Models for Automated Assessment of Breast Density Using Multiple Mammographic Image Types
Source: Cancers (Basel). 2022 Oct 13;14(20):5003. doi: 10.3390/cancers14205003 (PMC9599904; doi:10.3390/cancers14205003)
Supplement: Supplementary file 1 [file cancers-14-05003-s001.zip › cancers-1954862-supplementary.pdf]

Table S1. Participant demographics in training, validation, and test datasets of a total 5032 participants randomly separated in 3044, 993, and 995 subjects.

		Views	Training	Validation	Test
**Dataset size #**					
For processing mammography		R MLO =6939	14281	4399	8369
		R CC =6655			
		L MLO =6867			
		L CC =6588			
For presentation mammography		R-CC =10644	24356	7593	11756
		L MLO =10503			
		R MLO =11333			
		L CC =11225			
Synthesized 2D mammography (reconstructed from tomosynthesis)		R CC =6423	13972	4489	7415
		L MLO =6478			
		R MLO =6489			
		L CC =6486			
Digital breast tomosynthesis mammography		L CC =6268	14144	4538	6288
		L MLO =6225			
		R CC =6238			
		R MLO =6239			
For processing and presentation mammograms combined			14267	4396	8365
All modalities combined			12033	3712	5908
**Characteristics / Answers**					
Baseline BI-RADS Breast Density (year 0)	Fatty		104 (3.4%)	24 (2.4%)	36 (3.6%)
	Scattered Fibroglandular Densities		1265 (41.6%)	402 (40.5%)	390 (39.2%)

	Heterogeneously Dense	1556 (51.1%)	524 (52.8%)	535 (53.8%)
	Extremely Dense	115 (3.8%)	41 (4.1%)	33 (3.3%)
	Unknown	4 (0.1%)	2 (0.2%)	1 (0.1%)
Baseline BI-RADS Breast Density (year 1)	Fatty	62 (2%)	23 (2.3%)	34 (3.4%)
	Scattered Fibroglandular Densities	741 (24.3%)	225 (22.7%)	362 (36.4%)
	Heterogeneously Dense	911 (29.9%)	291 (29.3%)	462 (46.4%)
	Extremely Dense	81 (2.7%)	23 (2.3%)	31 (3.1%)
	Unknown	1249 (41.0%)	431 (43.4%)	106 (10.6%)
Baseline BI-RADS Breast Density (year 2)	Fatty	14 (0.5%)	6 (0.6%)	19 (1.9%)
	Scattered Fibroglandular Densities	229 (7.5%)	74 (7.4%)	276 (27.7%)
	Heterogeneously Dense	273 (9%)	92 (9.3%)	324 (32.6%)
	Extremely Dense	20 (0.6%)	8 (0.8%)	25 (2.5%)
	Unknown	2508 (82.4%)	813 (81.9%)	351 (35.3%)
Baseline BI-RADS Breast Density (year 3)	Fatty	0 (0%)	0 (0%)	4 (0.4%)
	Scattered Fibroglandular Densities	1 (0%)	2 (0.2%)	52 (5.2%)
	Heterogeneously Dense	1 (0%)	2 (0.2%)	57 (5.7%)
	Extremely Dense	1 (0%)	0 (0%)	5 (0.5%)
	Unknown	3041 (99.9%)	989 (99.6%)	877 (88.1%)
Age	Median (min-max)	58 (25-80)	58 (30-80)	57 (28-80)
	Missing	375 (12.3%)	119 (12%)	141 (14.2%)
Height (cm)	Median (min-max)	162 (125-188)	162 (130-182)	162 (127-186)
	Missing	552 (18.1%)	197 (19.8%)	9 (0.9%)
Weight (kg)	Median (min-max)	72.3 (39-183)	72 (40-145.3)	72.8 (45.7-150)
	Missing	550 (18.1)	197 (19.8)	9 (0.9%)

Menopausal status	Yes	2030 (67%)	664 (66.9%)	642 (64.5%)
	Irregular periods	112 (3.7%)	33 (3.3%)	36 (3.6%)
	No	660 (21.7%)	212 (21.3%)	237 (23.8%)
	Missing	242 (8%)	84 (8.4%)	80 (8%)
Racial background	American Indian / Alaska Native	10 (0.3%)	1 (0.1%)	4 (0.4%)
	Asian	193 (6%)	59 (5.9%)	54 (5.4%)
	Black, Hispanic	10 (0.3%)	5 (0.5%)	2 (0.2%)
	Black, Non-Hispanic	275 (9%)	87 (8.8%)	83 (8.3%)
	Native Hawaiian / Pacific Islander	4 (0.1%)	2 (0.2%)	2 (0.2%)
	Other	36 (1.2%)	10 (1%)	6 (0.6%)
	Undisclosed	6 (0.2%)	1 (0.1%)	3 (0.3%)
	White, Hispanic	351 (11.5%)	104 (10.5%)	97 (9.7%)
	White, Non-Hispanic	2013 (66.1%)	670 (67.5%)	697 (70%)
	Missing	146 (4.8%)	54 (5.4%)	47 (4.7%)
First-degree relatives diagnosed with Ovarian, Breast, or Pancreatic Cancer	Yes	969 (31.8%)	329 (33.1%)	357 (35.9%)
	No	1883 (61.9%)	594 (59.8%)	567 (57%)
	Don’t know	57 (1.9%)	23 (2.3%)	21 (2.1%)
	Missing	135 (4.4%)	47 (4.7%)	50 (5%)
Second-degree relatives diagnosed with Ovarian, Breast, or Pancreatic Cancer	Yes	1384 (45.5%)	441 (44.4%)	463 (46.5%)
	No	1221 (40.1%)	412 (41.5%)	379 (38.1%)
	Don’t know	293 (9.6%)	87 (8.7%)	97 (9.7%)
	Missing	146 (4.8%)	53 (5.3%)	56 (5.6%)
Smoker (>100 cigarettes in lifetime)	Yes, currently smoke	70 (2.3%)	15 (1.5%)	27 (2.7%)
	Yes, but I quit smoking	647 (21.2%)	225 (22.7%)	191 (19.2%)
	No, never	2152 (70.1%)	695 (70%)	717 (72.1%)
	Missing	175 (5.7%)	58 (5.8%)	60 (6%)

Age when starting smoking	Median (min-max)	18 (10-50)	17 (10-56)	18 (11-50)
	Missing	2336 (76.7%)	753 (75.8%)	777 (78.1%)
How many cigarettes per day?	Median (min-max)	10 (1-80)	10 (1-60)	8 (1-40)
	Missing	2346 (77.1%)	758 (76.3%)	781 (78.5%)
Age you when you quit smoking?	Median (min-max)	30 (14-73)	28.5 (16-65)	30 (15-67)
	Missing	2407 (79.1%)	769 (77.4%)	804 (80.8%)
Have you ever been exposed to other people's smoke on a daily or regular basis during childhood at home?	Rarely	1231 (40.4%)	415 (41.8%)	389 (39.1%)
	Regularly	1007 (33.1%)	306 (30.8%)	319 (32.1%)
	Sometimes	637 (20.1%)	212 (21.3%)	228 (22.9%)
	Missing	169 (5.5%)	60 (6%)	59 (5.9%)
Have you ever been exposed to other people's smoke on a daily or regular basis during adulthood at home?	Rarely	2082 (68.4%)	696 (70%)	699 (70.2%)
	Regularly	230 (7.5%)	66 (6.6%)	52 (5.2%)
	Sometimes	557 (18.3%)	163 (16.4%)	184 (18.5%)
	Missing	175 (5.7%)	68 (6.8%)	60 (6%)
Have you ever been exposed to other people's smoke on a daily or regular basis during adulthood at work?	Rarely	2245 (73.7%)	725 (73%)	731 (73.5%)
	Regularly	122 (4%)	37 (3.7%)	20 (2%)
	Sometimes	498 (16.3%)	163 (16.4%)	184 (18.5%)
	Missing	179 (5.9%)	68 (6.8%)	60 (6%)
Drink alcohol (>4 drinks per month during >6 months)	Yes, currently	1323 (43.5)	422 (42.5%)	482 (48.4%)
	Yes, but I quit	339 (11.1%)	123 (12.4%)	94 (9.4%)
	No, never	1214 (39.9%)	391 (39.4%)	355 (35.7%)
	Missing	168 (5.5%)	57 (5.7%)	64 (6.4%)

At what age did you first begin to drink alcohol on a regular basis?	Median (min-max)	21 (10-74)	21 (13-67)	21 (13-75)
	Missing	1428 (46.9%)	464 (46.7%)	433 (43.5)
Number of pregnancies	Median (min-max)	2 (0-11)	2 (0-8)	2 (0-12)
	Missing	250 (8.2%)	82 (8.3)	79 (7.9)
Regular physical activity	Often	656 (21.5%)	219 (22%)	224 (22.5%)
	Sometimes	1350 (44.3%)	437 (44%)	433 (43.5%)
	Never, rarely	773 (25.4%)	244 (24.6%)	245 (24.6%)
	Missing	265 (8.7%)	93 (9.4%)	93 (9.3%)
Birth control pills / hormones intakes (>3 months)	Current	157 (5.2%)	48 (4.8%)	39 (3.9%)
	Former	2155 (70.8%)	701 (70.6%)	751 (75.5%)
	Never	460 (15.1%)	114 (14.5%)	118 (11.9%)
	Don't know	48 (1.6%)	21 (2.1%)	10 (1%)
	Missing	224 (7.4%)	78 (8%)	77 (7.7%)
Have you ever used any replacement hormones (a pill or patch)? (excluding birth control pills and fertility drugs)	Currently	297 (9.8%)	115 (11.6%)	115 (11.6%)
	In the past	580 (19%)	177 (17.8%)	184 (18.5%)
	Never	1887 (62%)	607 (61.1%)	612 (61.5%)
	Don't know	55 (1.8%)	16 (1.6%)	7 (0.7%)
	Missing	225 (7.4%)	78 (7.8%)	77 (7.7%)
How old were you when you started your first menstrual period?	Median (min-max)	13 (8-45)	13 (9-18)	13 (7-45)
	Missing	513 (16.8%)	180 (18.1%)	164 (16.5%)

Table S2. Image parameters in training, validation and test datasets.

Image parameters	median (min-max)<br>missing # (missing %)		
**For processing mammography**	**Training**	**Validation**	**Test**
KVP<br>missing	31 (25-47)<br>18 (0%)	31 (25-46)<br>1 (0%)	31 (25-46)<br>8 (0%)
Exposure time (ms)<br>missing	1009 (10-2994)<br>18 (0%)	1039 (10-2976)<br>1 (0%)	1022 (10-4840)<br>8 (0%)
Xray tube current (mA)<br>missing	150 (40-200)<br>18 (0%)	150 (40-200)<br>1 (0%)	150 (0-200)<br>8 (0%)
Exposure (mAs)<br>missing	108 (0-502)<br>18 (0%)	112 (0-487)<br>1 (0%)	110 (0-466)<br>8 (0%)
Body part thickness (mm)<br>missing	63 (5-156)<br>18 (0%)	64 (16-146)<br>1 (0%)	64 (10-135)<br>9 (0%)
Compression force (N)<br>missing	87 (0-230)<br>18 (0%)	87 (0-240)<br>1 (0%)	86 (0-270)<br>15 (0%)
Relative Xray exposure<br>missing	514 (0-5598)<br>18 (0%)	515 (0-7057)<br>1 (0%)	512 (0-4237)<br>9 (0%)
Image rows<br>missing	3328 (425-4096)<br>0 (0%)	3328 (550-4096)<br>0 (0%)	3328 (480-4096)<br>0 (0%)
Image columns<br>missing	2560 (266-3328)<br>0 (0%)	2560 (880-3328)<br>0 (0%)	2560 (750-3328)<br>0 (0%)
Pixel spacing (mm)<br>missing	0.065 (0.04-0.11)<br>70 (0.1%)	0.065 (0.04-0.11)<br>10 (0%)	0.065 (0.02-0.011)<br>15 (0%)

Organ dose (dGy)	0.02 (0-0.09)	0.02 (0-0.1)	0.02 (0-0.08)
missing	20497 (22.3%)	6597 (22.3%)	8092 (21.9%)
**For presentation mammography**	**Training**	**Validation**	**Test**
KVP	31 (25-47)	31 (25-46)	31 (25-46)
missing	18 (0%)	1 (0%)	8 (0%)
Exposure time (ms)	1009 (10-2994)	1039 (10-2976)	1022 (10-4840)
missing	18 (0%)	1 (0%)	8 (0%)
Xray tube current (mA)	150 (40-200)	150 (40-200)	150 (0-200)
missing	18 (0%)	1 (0%)	8 (0%)
Exposure (mAs)	108 (0-502)	112 (0-487)	110 (0-466)
missing	18 (0%)	1 (0%)	8 (0%)
Body part thickness (mm)	63 (5-156)	64 (16-146)	64 (10-135)
missing	18 (0%)	1 (0%)	9 (0%)
Compression force (N)	87 (0-230)	87 (0-240)	86 (0-270)
missing	18 (0%)	1 (0%)	15 (0%)
Relative Xray exposure	514 (0-5598)	515 (0-7057)	512 (0-4237)
missing	18 (0%)	1 (0%)	9 (0%)
Image rows	3328 (425-4096)	3328 (550-4096)	3328 (480-4096)
missing	0 (0%)	0 (0%)	0 (0%)
Image columns	2560 (266-3328)	2560 (880-3328)	2560 (750-3328)
missing	0 (0%)	0 (0%)	0 (0%)
Pixel spacing (mm)	0.065 (0.04-0.11)	0.065 (0.04-0.11)	0.065 (0.02-0.011)
missing	70 (0.1%)	10 (0%)	15 (0%)
Organ dose (dGy)	0.02 (0-0.09)	0.02 (0-0.1)	0.02 (0-0.08)
missing	20497 (22.3%)	6597 (22.3%)	8092 (21.9%)

**Synthesized 2D mammography (reconstructed from tomosynthesis)**	**Training**	**Validation**	**Test**
KVP	31 (25-47)	31 (25-46)	
missing	18 (0%)	1 (0%)	31 (25-46) 8 (0%)
Exposure time (ms)	1009 (10-2994)	1039 (10-2976)	1022 (10-4840)
missing	18 (0%)	1 (0%)	8 (0%)
Xray tube current (mA)	150 (40-200)	150 (40-200)	150 (0-200)
missing	18 (0%)	1 (0%)	8 (0%)
Exposure (mAs)	108 (0-502)	112 (0-487)	110 (0-466)
missing	18 (0%)	1 (0%)	8 (0%)
Body part thickness (mm)	63 (5-156)	64 (16-146)	64 (10-135)
missing	18 (0%)	1 (0%)	9 (0%)
Compression force (N)	87 (0-230)	87 (0-240)	86 (0-270)
missing	18 (0%)	1 (0%)	15 (0%)
Relative Xray exposure	514 (0-5598)	515 (0-7057)	512 (0-4237)
missing	18 (0%)	1 (0%)	9 (0%)
Image rows	3328 (425-4096)	3328 (550-4096)	3328 (480-4096)
missing	0 (0%)	0 (0%)	0 (0%)
Image columns	2560 (266-3328)	2560 (880-3328)	2560 (750-3328)
missing	0 (0%)	0 (0%)	0 (0%)
Pixel spacing (mm)	0.065 (0.04-0.11)	0.065 (0.04-0.11)	0.065 (0.02-0.011)
missing	70 (0.1%)	10 (0%)	15 (0%)
Organ dose (dGy)	0.02 (0-0.09)	0.02 (0-0.1)	0.02 (0-0.08)
missing	20497 (22.3%)	6597 (22.3%)	8092 (21.9%)
**Digital breast tomosynthesis mammography**	**Training**	**Validation**	**Test**

KVP	31 (25-47)	31 (25-46)	31 (25-46)
missing	18 (0%)	1 (0%)	8 (0%)
Exposure time (ms)	1009 (10-2994)	1039 (10-2976)	1022 (10-4840)
missing	18 (0%)	1 (0%)	8 (0%)
Xray tube current (mA)	150 (40-200)	150 (40-200)	150 (0-200)
missing	18 (0%)	1 (0%)	8 (0%)
Exposure (mAs)	108 (0-502)	112 (0-487)	110 (0-466)
missing	18 (0%)	1 (0%)	8 (0%)
Body part thickness (mm)	63 (5-156)	64 (16-146)	64 (10-135)
missing	18 (0%)	1 (0%)	9 (0%)
Compression force (N)	87 (0-230)	87 (0-240)	86 (0-270)
missing	18 (0%)	1 (0%)	15 (0%)
Relative Xray exposure	514 (0-5598)	515 (0-7057)	512 (0-4237)
missing	18 (0%)	1 (0%)	9 (0%)
Image rows	3328 (425-4096)	3328 (550-4096)	3328 (480-4096)
missing	0 (0%)	0 (0%)	0 (0%)
Image columns	2560 (266-3328)	2560 (880-3328)	2560 (750-3328)
missing	0 (0%)	0 (0%)	0 (0%)
Image slices	64(42-89)	64(42-89)	64(42-89)
	0 (0%)	0 (0%)	0 (0%)
Pixel spacing (mm)	0.065 (0.04-0.11)	0.065 (0.04-0.11)	0.065 (0.02-0.011)
missing	70 (0.1%)	10 (0%)	15 (0%)
Organ dose (dGy)	0.02 (0-0.09)	0.02 (0-0.1)	0.02 (0-0.08)
missing	20497 (22.3%)	6597 (22.3%)	8092 (21.9%)

Table S3. Model accuracy on their respective validation dataset for all screening years for 4 BI-RADS categories.

Model	Include history	Metric										
		TP	FP	Cat. Acc.	Precision	Recall	AUC	F1 per class				Kappa
								1	2	3	4	
**History**	**N/A**	611	376	0.62	0.58	0.62	0.69	0.00	0.55	0.70	0.00	0.26
**For processing mammography**	**No**	813	171	0.83	0.81	0.83	0.93	0.12	0.82	0.87	0.57	0.68
	**Yes**	812	172	0.83	0.83	0.83	0.93	0.07	0.83	0.86	0.48	0.67
**For presentation mammography**	**No**	1378	248	0.85	0.84	0.85	0.95	0.32	0.86	0.88	0.46	0.72
	**Yes**	1374	252	0.85	0.84	0.85	0.94	0.28	0.85	0.88	0.44	0.71
**Synthesized 2D mammography**	**No**	960	175	0.85	0.83	0.85	0.94	0.07	0.85	0.88	0.57	0.71
	**Yes**	955	180	0.84	0.83	0.84	0.94	0.18	0.84	0.88	0.55	0.70
**Digital breast tomosynthesis**	**No**	968	179	0.84	0.84	0.84	0.94	0.18	0.84	0.88	0.58	0.71
	**Yes**	964	183	0.84	0.83	0.84	0.94	0.26	0.84	0.88	0.52	0.70
**All modality**	**No**	705	123	0.85	0.85	0.85	0.94	0.10	0.84	0.89	0.60	0.72
	**Yes**	706	122	0.85	0.84	0.85	0.94	0.08	0.85	0.89	0.60	0.72

Table S4. Model accuracy on their respective validation dataset for all screening years for 2 categories (Non-dense and dense).

Model	Include history	TP	FP	Cat. Acc.	Precision	Recall	AUC	F1 per class: Non-dense	F1 per class: Dense	Kappa
**History**	**N/A**	671	316	0.68	0.68	0.68	0.73	0.60	0.73	0.33
**For processing mammography**	**No**	868	116	0.88	0.88	0.88	0.96	0.86	0.90	0.76
	**Yes**	868	116	0.88	0.88	0.88	0.96	0.86	0.90	0.76
**For presentation mammography**	**No**	1473	153	0.91	0.91	0.91	0.97	0.89	0.92	0.81
	**Yes**	1468	158	0.90	0.90	0.90	0.97	0.89	0.91	0.80
**Synthesized 2D mammography**	**No**	1017	118	0.90	0.90	0.90	0.96	0.88	0.91	0.79
	**Yes**	1015	120	0.89	0.89	0.89	0.96	0.88	0.91	0.78
**Digital breast tomosynthesis**	**No**	1031	116	0.90	0.90	0.90	0.96	0.88	0.91	0.79
	**Yes**	1029	118	0.90	0.90	0.90	0.96	0.88	0.91	0.79
**All modality**	**No**	742	86	0.90	0.90	0.90	0.96	0.88	0.91	0.79
	**Yes**	750	78	0.91	0.91	0.91	0.96	0.89	0.92	0.81

Figure S1. Confusion matrix for binary breast density for each model on every screening year of the test dataset without including patient history for A) history, B) for processing, C) for presentation, D) Synthesized 2D, E) digital breast tomosynthesis, mammograms and F) all modalities models.

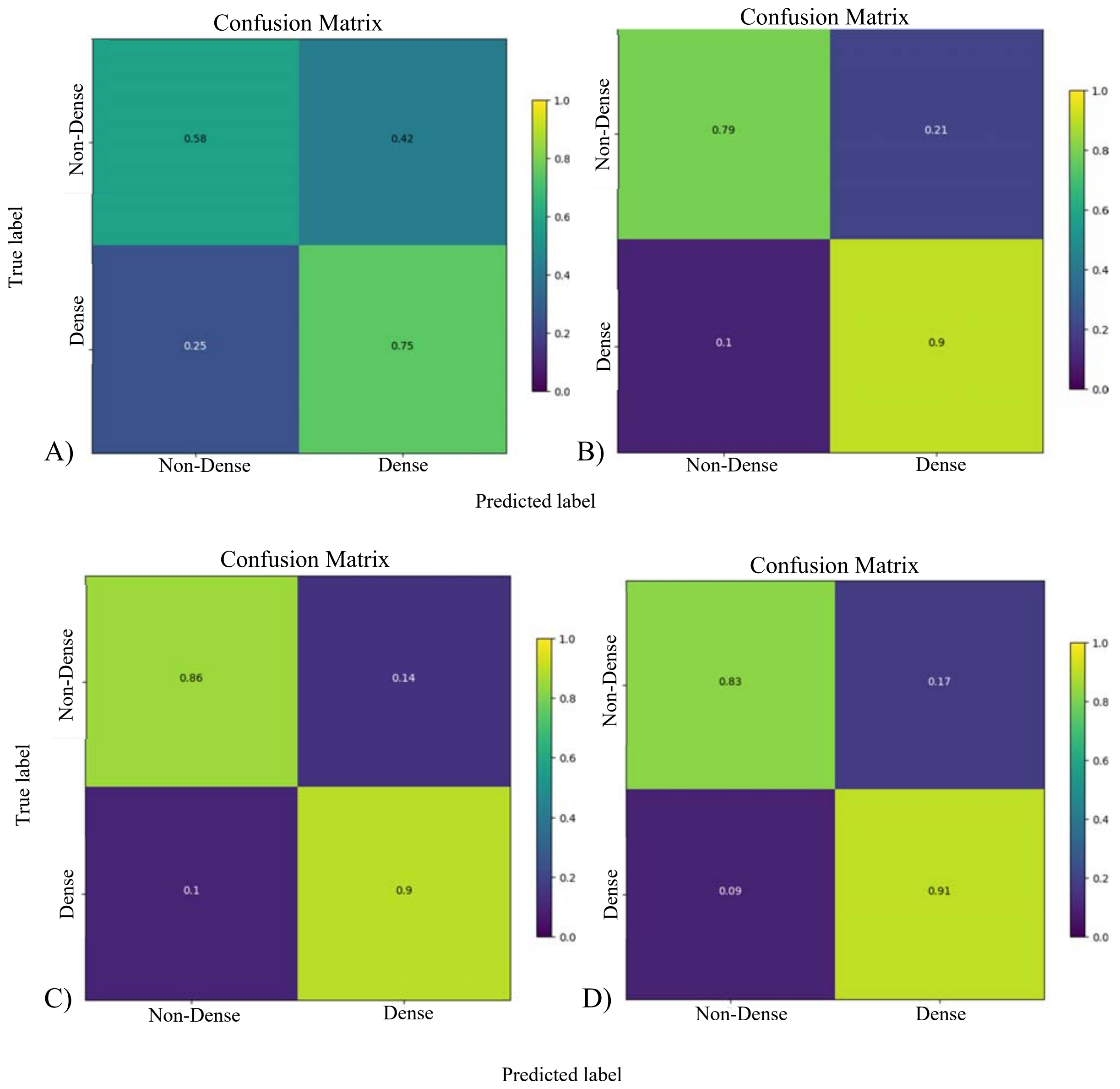

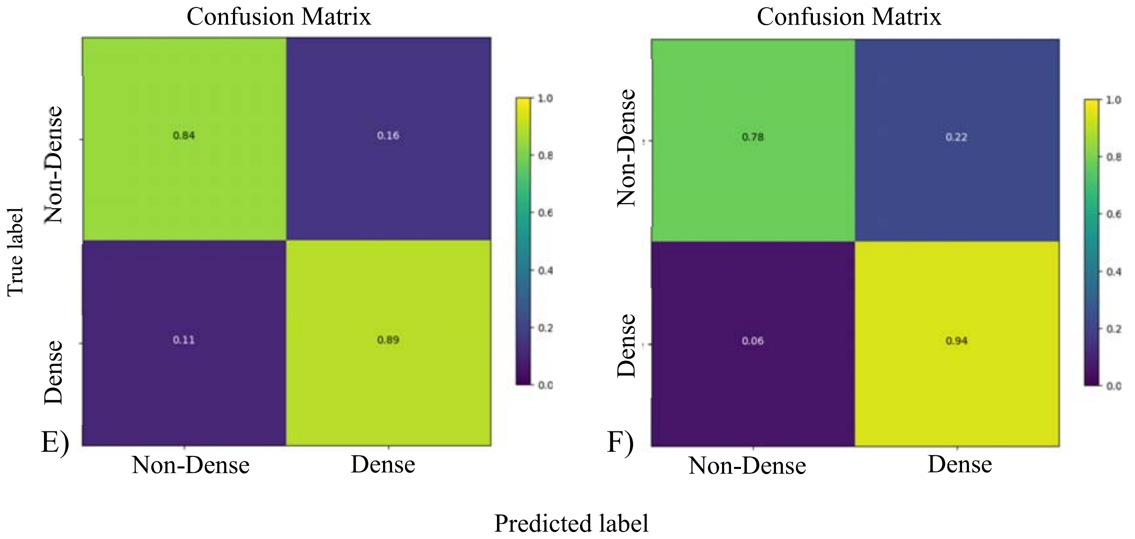
Confusion Matrix
Confusion Matrix
True label
Non-Dense
Dense
0.84
0.16
0.11
0.89
E)
Non-Dense
Dense
Non-Dense
Dense
0.78
0.22
0.06
0.94
F)
Non-Dense
Dense
Predicted label
1.0
0.8
0.6
0.4
0.2
0.0

Figure S2. Distribution of breast density category assignment for the ground truth and DL models on for A) 4 categories and B) 2 categories, on the validation dataset considering every screening (except H).

Abbreviations: GT: ground truth (reader consensus), H: history model, R1: for processing, M1: for presentation, CV1: Synthesized 2D, TM1: digital breast tomosynthesis, MM1: Multi model, 2: include patient history.

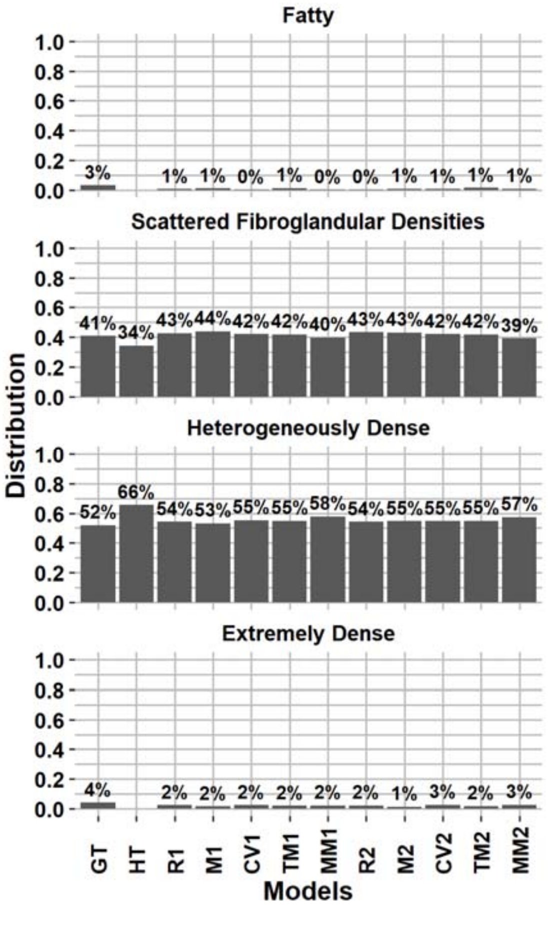

A)

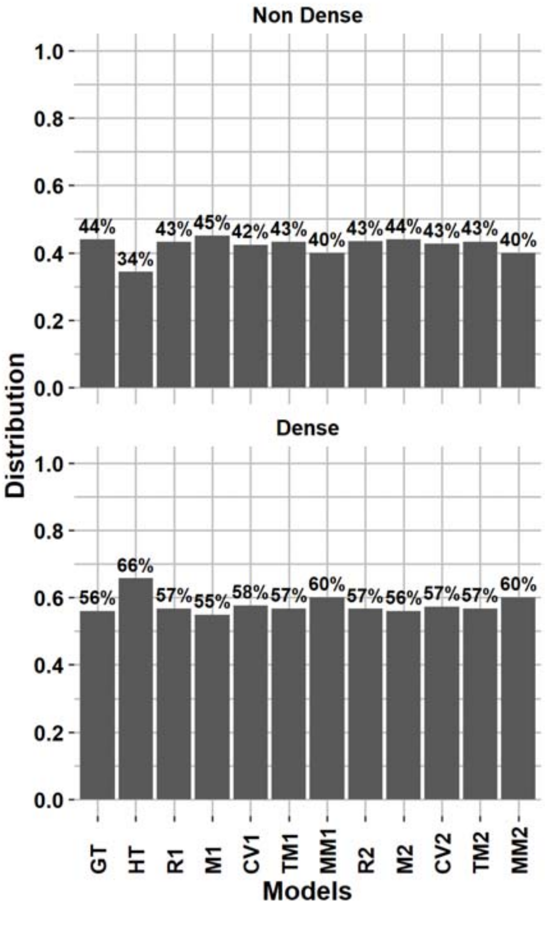

B)

Figure S3. Distribution of breast density category assignment for the ground truth and DL models on for A) 4 categories and B) 2 categories, on the test dataset considering the year 0 screening.

Abbreviations: GT: ground truth (reader consensus), H: history model, R1: for processing, M1: for presentation, CV1: Synthesized 2D, TM1: digital breast tomosynthesis, MM1: Multi model, 2: include patient history.

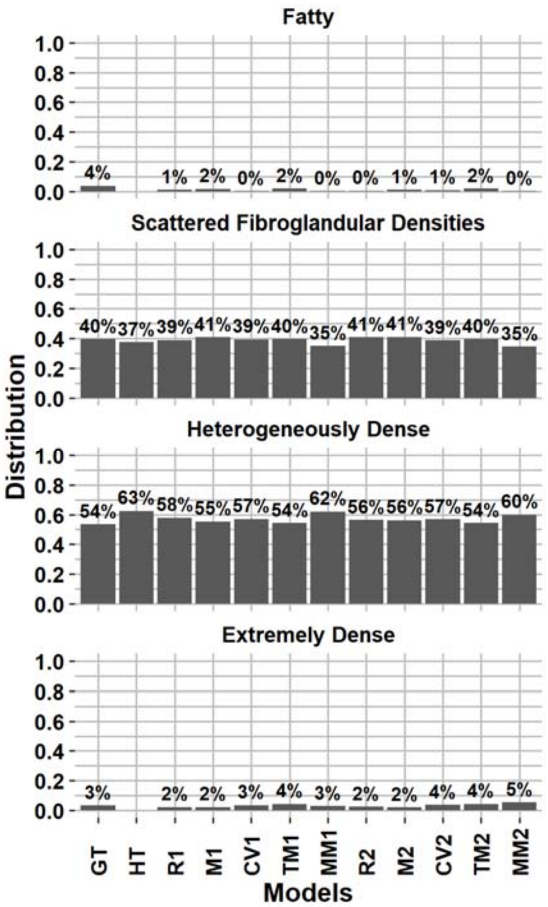

A)

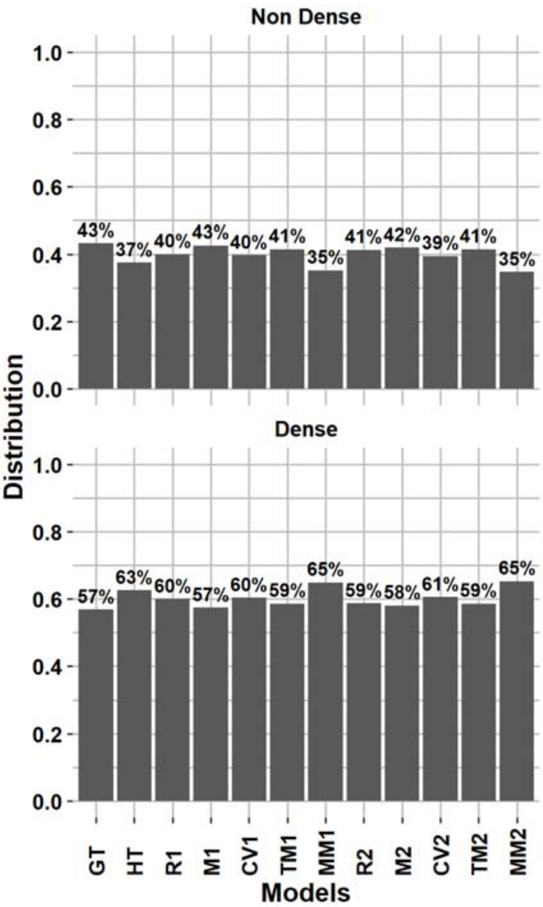

B)
